# Association of leukocyte cell-derived chemotaxin 2 (LECT2) with NAFLD, metabolic syndrome, and atherosclerosis

**DOI:** 10.1371/journal.pone.0174717

**Published:** 2017-04-04

**Authors:** Hye Jin Yoo, Soon Young Hwang, Ju-Hee Choi, Hyun Jung Lee, Hye Soo Chung, Ji-A Seo, Sin Gon Kim, Nan Hee Kim, Sei Hyun Baik, Dong Seop Choi, Kyung Mook Choi

**Affiliations:** 1 Division of Endocrinology and Metabolism, Department of Internal Medicine, College of Medicine, Korea University, Seoul, Korea; 2 Department of Biostatistics, College of Medicine, Korea University, Seoul, Korea; Medizinische Fakultat der RWTH Aachen, GERMANY

## Abstract

**Objective:**

Previous studies have shown that leukocyte cell-derived chemotaxin 2 (LECT2), a recently discovered hepatokine, is associated with the inflammatory response and insulin resistance. We examined circulating plasma LECT2 levels in the subjects with non-alcoholic fatty liver disease (NAFLD) or metabolic syndrome.

**Methods:**

We analyzed plasma LECT2 levels from the subjects of age- and sex-matched 320 adults with or without NAFLD who completed a health check-up at the Health Promotion Center of Korea University Guro Hospital.

**Results:**

Individuals with NAFLD showed significantly higher LECT2 levels (31.2 [20.9, 41.5] vs. 24.5[16.3, 32.7] ng/ml, *P* <0.001) as well as components of MetS compared to those without NAFLD. Furthermore, circulating LECT2 concentrations were greater in subjects with MetS (32.6 [17.8, 45.0] vs. 27.0 [18.7, 33.7] ng/ml, *P* = 0.016) and were associated with anthropometric measures of obesity, lipid profiles, high sensitivity C-reactive protein (hsCRP) and liver aminotransferase levels. However, there was no significant relationship between LECT2 levels and indicators of subclinical atherosclerosis, such as carotid intima-media thickness (CIMT) and brachial ankle pulse wave velocity (baPWV). Multivariate analysis demonstrated a progressively increasing trend of odds ratios for NAFLD according to quartiles of LECT2 levels after adjusting for risk factors, although the relationship was attenuated after further adjustment for waist circumference and lipid levels.

**Conclusion:**

Circulating LECT2 concentrations were increased in individuals with NAFLD and those with MetS, but not in those with atherosclerosis. The relationship between LECT2 and both NAFLD and MetS might be mediated by its association with abdominal obesity and lipid metabolism.

**Trial registration:**

Clinicaltrials.gov NCT01594710

## Introduction

Non-alcoholic fatty liver disease (NAFLD) causes up to 33% of chronic liver disease worldwide, more than any other cause [1]. NAFLD, which has been considered a hepatic manifestation of metabolic syndrome, increases the risk of developing cardiovascular disease (CVD) and type 2 diabetes mellitus independently from the traditional risk factors [2]. The liver itself releases a variety of proatherogenic, proinflammatory, and diabetogenic mediators such as high-sensitivity C-reactive protein (hsCRP), fibrinogen, and plasminogen activator inhibitor-1 (PAI-1) in subjects with NAFLD [3]. Recent studies have shown that hepatokines, predominantly liver-derived and secreted proteins, directly affect glucose and lipid metabolism in a way similar to adipokines and myokines [4,5].

Leukocyte cell-derived chemotaxin 2 (LECT2) is a 16-kDa secretory protein that was first isolated in 1996 from cultured supernatants of phytohemagglutinin-activated human T-cell leukemia SKW-3 cells [6]. Initially, it was identified as a chemotactic factor for neutrophils [6]. Later, Lu *et al*. [7] showed that LECT2 treatment in mice significantly increased the number of infiltrating polymorphonuclear neutrophils and macrophages into the peritoneum after *Escherichia coli* injection. Furthermore, LECT2 was involved in the pathogenesis of hepatitis by modulation of natural killer T-cells [8]. Ovejero *et al*. [9] reported that β-catenin-induced LECT2 expression inhibits tumor progression in the liver by suppressing inflammatory and immune responses. Although growing evidence suggests that LECT2 acts as a modulator of immune and inflammatory reactions, few studies have focused on the function of LECT2 in metabolic disorders. Recently, Lan *et al*. [10] redefined LECT2 as a novel hepatokine that mediates obesity with skeletal muscle insulin resistance. In that study, genetic deletion of LECT2 in mice increased insulin sensitivity in skeletal muscle, but treatment with recombinant LECT2 protein impaired insulin signaling via phosphorylation of Jun NH2-terminal kinase (JNK) in myocytes. They also showed that the expression of the genes involved in mitochondria and myogenesis was up-regulated in the muscle of LECT^-/-^ mice [10], emphasizing the pivotal role of LECT2 on the provocation of peripheral insulin resistance. Recently, we reported that a dipeptidyl peptidase 4 inhibitor improves hepatic steatosis as well as insulin resistance through AMP-activated protein kinase (AMPK)- and JNK-dependent inhibition of LECT2 expression [11]. Furthermore, we found that LECT2 induces pro-inflammatory cytokines and adhesion molecules via CD209 receptor-mediated JNK phosphorylation in human umbilical vein endothelial cells (HUVECs) [12]. However, there have been very limited studies to examine the clinical significance of circulating LECT2 levels in metabolic diseases including NAFLD and atherosclerosis in humans.

Therefore, we evaluated whether circulating levels of LECT2 are increased in the subjects with NAFLD and exhibit significant correlations with various components of metabolic syndrome as well as systemic low-grade inflammatory status, represented as high sensitivity C-reactive protein (hsCRP) and subclinical atherosclerosis measured using carotid intima-media thickness (CIMT) and brachial-ankle pulse wave velocity (baPWV).

## Subjects and methods

### Study design and participants

Our group had collected the anthropometric and laboratory data as well as blood samples from participants among individuals who were self-referred for a routine health check-up at the Health Promotion Center of Korea University Guro Hospital, between April 2012 and July 2014. All participants provided written informed consent, and the Korea University Institutional Review Board approved this study protocol in accordance with the Declaration of Helsinki of the World Medical Association. From the baseline samples of these cohort subjects (n = 1,128), we randomly selected NAFLD group (n = 160) and age, sex-matched control group (n = 160) based on the inclusion and exclusion criteria. We had included participants who are agreed to participate in our study and subjects had been excluded if they met any of the following criteria: history of CVD (myocardial infarction, unstable angina, stroke, or cardiovascular revascularization); stage 2 hypertension (resting blood pressure, ≥160/100 mmHg); history of inflammatory conditions that could affect the study results; taking medications that might affect inflammatory status including steroid and non-steroidal anti-inflammatory drugs within six months; hepatic malignancy; or severe renal or hepatic disease. To avoid cofounding variables associated with NAFLD, participants were additionally excluded for this study if they exhibited: consumption of >140 g alcohol/week [13], a positive test for hepatitis B surface antigen or hepatitis C antibody, or use of herbal medication within 6 months. In this study, NAFLD was diagnosed on ultrasonography by a single experienced radiologist who was blinded to the anthropometric and laboratory data. Metabolic syndrome was defined according to criteria established by the National Cholesterol Education Program Adult Treatment Panel III (NCEP III) using the adjusted waist circumference for Asians [14]. The criteria require the presence of three or more of the following components: 1) high blood pressure (systolic blood pressure ≥ 130, diastolic blood pressure ≥85 mmHg, or known treatment for hypertension), 2) hypertriglyceridemia (fasting plasma triglycerides ≥1.69 mmol/L), 3) low high density lipoprotein cholesterol (HDL-C) (fasting HDL-C <1.04 mmol/L in men, <1.29 mmol/L in women), and 4) hyperglycemia (fasting plasma glucose ≥5.6 mmol/L or known treatment for diabetes). Medical histories and lifestyle information were collected for all subjects by personal interview using a detailed questionnaire.

### Anthropometric and laboratory measurements

Body mass index (BMI) was calculated as weight/height^2^ (kg/m^2^) and waist circumference was measured at the midpoint between the lower border of the rib cage and the iliac crest. All blood samples were obtained in the morning after a 12-hour overnight fast, and were immediately stored at -80°C for subsequent analyses. Serum triglyceride and HDL-C levels were determined enzymatically using a model 747 chemistry analyzer (Hitachi, Tokyo, Japan). The glucose oxidase method was used to measure plasma glucose levels. Latex-enhanced turbidimetric immunoassay (HiSens hsCRP LTIA; HBI, Anyang, Korea) was used for measurement of hsCRP with an inter-assay coefficient of variation of 7.2%. Plasma LECT2 levels were assayed using a commercially available ELISA (MBL, Nagoya, Japan); the intra-assay variations were 2.2–3.2%

### Measurement of baPWV

After subjects rested in the supine position for 5 minutes, baPWV was measured using a volume-plethysmographic apparatus (model BP-203RPE II; Colin, Komaki, Japan) that simultaneously recorded baPWV and brachial and ankle blood pressures on the left and right sides. The details of this method, including validity and reproducibility, were described previously [15]. The intra- and inter-observer reproducibility of this method in the present study was 10.0% and 8.4%, respectively. The baPWV was calculated as the mean of the left and right baPWV values.

### Measurement of carotid IMT

The IMT of the common carotid artery was determined using high-resolution B-mode ultrasonography (EnVisor; Philips Medical Systems, Andover, MA, USA) with a 5-12-MHz transducer. Measurements of the carotid IMT were made using IMT measurement software (Intimascope; Media Cross Co., Tokyo, Japan) at three levels of the lateral walls of the carotid artery, 1–3 cm proximal to the carotid bifurcation. The mean carotid IMT was the mean value of the mean left and right IMT measurements.

### Statistical analysis

We performed the Shapiro–Wilk test to evaluate the normality of continuous variables. As a result of normality test, all data are expressed as a median (inter-quartile range [25%, 75%]) for continuous variables or N (%) for categorical variables. Differences among the groups were tested using the Mann-Whitney test for continuous variables or Pearson’s chi-square test for categorical variables. Spearman’s correlation coefficient was used to evaluate the correlations of serum LECT2 levels with metabolic risk factors. Multiple logistic regression analysis was performed to identify whether quartiles of circulating LECT2 levels could influence the risk of NAFLD even after adjusting for other confounding factors. All statistical results were based on two-sided tests. Data was analyzed using SAS 9.4 (SAS Institute, Cary, NC, USA). P-values <0.05 were considered statistically significant.

## Results

### Baseline characteristic of subjects with or without NAFLD

**Fig 1:**
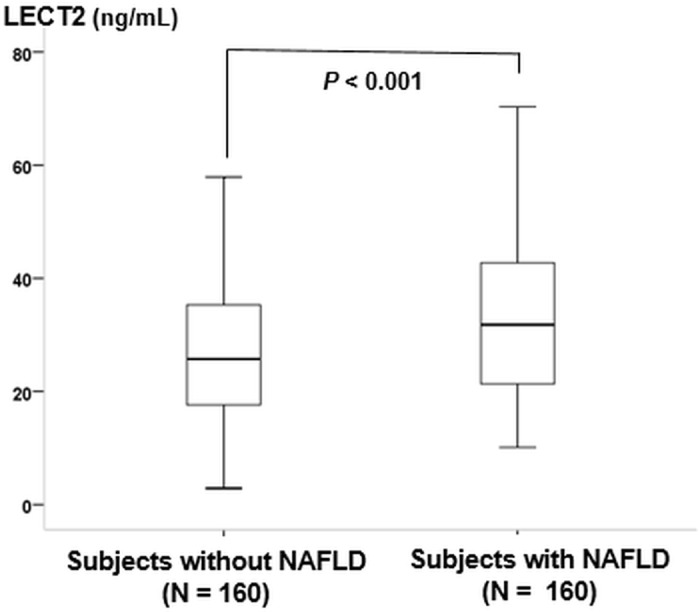
Comparison of circulating leukocyte cell-derived chemotaxin 2 (LECT2) concentrations in the subjects with Non-Alcoholic Fatty Liver Disease (NAFLD) vs. those without NAFLD (31.2 [20.9, 41.5] vs. 24.5 [16.3, 32.7] ng/ml, *P* <0.001). *P*-values represent the results of a Mann-Whitney test that was conducted to detect differences between the two groups.

The clinical and biochemical characteristics of the study subjects are presented in Table 1. Subjects with NAFLD showed a significantly higher BMI, waist circumference, systolic blood pressure, white blood cell (WBC) count, albumin concentration, aspartate aminotransferase (AST) and alanine aminotransferase (ALT) concentration, hsCRP, fasting glucose level, and triglyceride level compared to the control group. In contrast, HDL-C level in the NAFLD group was significantly lower than in the control group. In particular, plasma LECT2 levels in the NAFLD group were significantly higher than the subjects without NAFLD (31.2 [20.9, 41.5] vs. 24.5[16.3, 32.7] ng/ml, *P* <0.001) (Fig 1).

**Table 1:** Baseline characteristics of study subjects.

	Subjects without NAFLD (n = 160)	Subjects with NAFLD (n = 160)	*P*
Age (years)	52.5 (46.5, 56.5)	52.5 (47.0, 57.0)	0.910
Sex (M:F)	104:56	104:56	1.000
BMI (kg/m^2^)	23.1 (21.5, 25.0)	25.6 (23.9, 27.9)	<0.001
Waist circumference (cm)	78 (72.5, 84)	85 (81, 91)	<0.001
Current smoker (%)	36 (22.5)	36 (22.5)	1.000
Alcohol consumption (%)	95 (59.4)	79 (49.4)	0.073
Systolic BP (mmHg)	116 (106, 127)	118 (111, 129.5)	0.016
Diastolic BP (mmHg)	76 (70, 84)	78 (70, 87)	0.184
Hemoglobin (g/dL)	14.4 (13.1, 15.1)	14.4 (13.5, 15.3)	0.246
WBC (10^3^/μL)	5.4 (4.4, 6.5)	6.0 (5.2, 7.0)	<0.001
Platelet count (10^3^/μL)	204 (181.0, 237.5)	220.5 (188.5, 245.0)	0.028
BUN (mg/dL)	13.7 (11.9, 16.6)	15.1 (12.5, 17.4)	0.045
Creatinine (mg/dL)	0.78 (0.62, 0.86)	0.79 (0.64, 0.91)	0.159
Total bilirubin (mg/dL)	0.74 (0.61, 0.99)	0.72 (0.56, 0.93)	0.373
Albumin (g/dL)	4.4 (4.2, 4.5)	4.4 (4.3, 4.5)	0.001
AST (IU/L)	23 (20, 31)	27 (22, 37)	<0.001
ALT (IU/L)	19 (15, 26)	30 (20, 42)	<0.001
hsCRP (mg/dL)	0.5 (0.2, 1.4)	0.9 (0.4, 2.2)	0.003
Glucose (mmol/L)	5.22 (4.94, 5.61)	5.55 (5.19, 6.05)	<0.001
LDL cholesterol (mmol/L)	3.15 (2.64, 3.83)	3.10 (2.59, 3.85)	0.703
HDL cholesterol (mmol/L)	1.40 (1.16, 1.73)	1.22 (1.03, 1.40)	<0.001
Triglyceride (mmol/L)	0.99 (0.67, 1.54)	1.57 (1.03, 2.25)	<0.001
Mean baPWV (m/sec)	13.11 (12.01, 14.35)	13.34 (12.38, 14.75)	0.171
Mean CIMT (mm)	0.65 (0.58, 0.72)	0.67 (0.61, 0.73)	0.061

NAFLD, non-alcoholic fatty liver disease; BMI, body mass index; BP, blood pressure; WBC, white blood cell; BUN, blood urea nitrogen; AST, aspartate transaminase; ALT, alanine transaminase; hsCRP, high-sensitivity C-reactive protein; LDL, low-density lipoprotein; HDL, high-density lipoprotein; baPWV, brachial-ankle pulse wave velocity; CIMT, carotid intima-media thickness. *P*-values represent the results of a Mann-Whitney test or Pearson’s Chi-square test, which were conducted to detect differences between the two groups.

### Correlation of circulating LECT2 concentrations with metabolic risk factors

**Fig 2:**
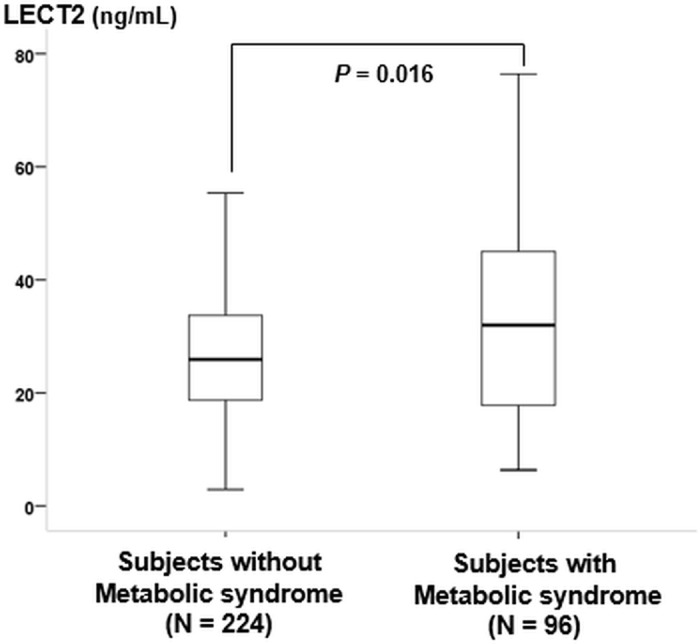
Comparison of circulating leukocyte cell-derived chemotaxin 2 (LECT2) concentrations in the subjects with metabolic syndrome (n = 96) vs. those without metabolic syndrome (n = 224) (32.6 [17.8, 45.0] vs. 27.0 [18.7, 33.7] ng/ml, *P* = 0.016). *P*-values represent the results of a Mann-Whitney test that was conducted to detect differences between the two groups.

Plasma LECT2 levels had significant positive correlations with BMI, waist circumference, hemoglobin, creatinine, albumin, AST, ALT, LDL cholesterol (LDL-C), triglyceride and hsCRP levels and a significant negative correlation with HDL-C in the total subjects (Table 2). In addition, circulating LECT2 level had a significant positive correlation with the number of metabolic syndrome components in the total subjects. Interestingly, although circulating LECT2 concentrations were significantly correlated with various metabolic risk factors in the subjects with NAFLD, plasma LECT2 levels were not significantly correlated with most of the metabolic risk factors in subjects without NAFLD; two exceptions were serum albumin and ALT levels. Furthermore, plasma LECT2 levels exhibited no significant association with mean CIMT or baPWV values both in control and NAFLD groups. We reclassified total subjects into the subjects with metabolic syndrome (n = 96) and without metabolic syndrome (n = 224). As a result, LECT2 concentrations in the subjects with metabolic syndrome were significantly higher than the subjects without metabolic syndrome (32.6 [17.8, 45.0] vs. 27.0 [18.7, 33.7] ng/ml, *P* = 0.016) (Fig 2).

**Table 2:** Spearman correlation coefficients for the relationship between circulating LECT2 levels and various metabolic risk factors.

	Total		Subjects without NAFLD		Subjects with NAFLD	
	*r*	*P*	*R*	*P*	*r*	*P*
Age	-0.09	0.103	-0.08	0.307	-0.11	0.156
Sex	0.07	0.225	0.00	0.983	0.15	0.061
BMI	0.23	<.0001	0.14	0.073	0.18	0.024
Waist circumference	0.24	<.0001	0.11	0.157	0.21	0.007
Systolic BP (mmHg)	0.09	0.090	-0.03	0.748	0.16	0.041
Diastolic BP (mmHg)	0.11	0.056	0.00	0.973	0.18	0.020
Hemoglobin	0.15	0.009	0.12	0.134	0.17	0.030
WBC	0.06	0.318	0.01	0.855	0.00	0.952
Platelet count	0.02	0.686	-0.01	0.942	0.01	0.900
BUN	0.05	0.345	0.08	0.307	-0.02	0.797
Creatinine	0.15	0.008	0.11	0.163	0.15	0.051
Total bilirubin	0.05	0.337	0.05	0.531	0.08	0.308
Albumin	0.17	0.003	0.22	0.006	0.06	0.460
AST	0.12	0.037	0.1	0.231	0.04	0.585
ALT	0.19	0.001	0.19	0.015	0.03	0.731
hsCRP	0.16	0.004	0.06	0.448	0.20	0.009
Glucose	0.07	0.204	-0.06	0.444	0.08	0.306
LDL cholesterol	0.12	0.035	0.04	0.592	0.20	0.013
HDL cholesterol	-0.17	0.002	-0.08	0.333	-0.16	0.050
Triglycerides	0.16	0.003	0.03	0.720	0.15	0.060
Number of MetS components	0.13	0.018	-0.05	0.551	0.15	0.055
Mean baPWV	0.02	0.745	-0.03	0.665	0.03	0.704
Mean CIMT	0.04	0.461	-0.01	0.934	0.04	0.645

NAFLD, non-alcoholic fatty liver disease; BMI, body mass index; BP, blood pressure; WBC, white blood cell; BUN, blood urea nitrogen; AST, aspartate transaminase; ALT, alanine transaminase; hsCRP, high-sensitivity C-reactive protein; LDL, low-density lipoprotein; HDL, high-density lipoprotein; MetS, metabolic syndrome; baPWV, brachial-ankle pulse wave velocity; CIMT, carotid intima-media thickness. *P*-values represent the results of Spearman’s correlation analysis.

### The odds ratio for NAFLD according to quartile of circulating LECT2 levels

Univariate and multivariate logistic regression analysis was used to assess the odd ratios (OR) of NAFLD across the quartiles of plasma LECT2 concentrations (Table 3). In an unadjusted model, the OR for NAFLD was 2.51 (95% CI, 1.22–5.19) in the highest quartile of plasma LECT2 levels when compared to that of the lowest. After controlling for age, sex, AST, ALT, total bilirubin, systolic blood pressure, serum glucose and hsCRP levels (model 3), the significance of the association between plasma LECT2 levels and NAFLD persisted (*P* for linear trend = 0.028); the OR in the highest quartile of circulating LECT2 level for NAFLD was 2.38 (95% CI, 1.06–5.37) when compared to that of the lowest. However, after further adjustment for serum triglyceride, HDL-C, LDL-C levels and waist circumference (model 5), the statistical significance between circulating LECT2 and NAFLD was attenuated (*P* for linear trend = 0.165).

**Table 3:** Odds ratios for NAFLD according to circulating LECT2 quartiles.

	Q1	Q2	Q3	Q4	*P* for linear trend
Number of cases/references	15/65	15/65	21/60	29/50	
LECT2 (range, ng/ml)	2.89–18.68	18.70–27.21	27.29–36.80	37.46–76.42	
Univariate	1	1.00 (0.45, 2.21)	1.52 (0.72, 3.21)	2.51 (1.22, 5.19)	0.006
Multivariate					
Model 1	1	1.00 (0.45, 2.22)	1.52 (0.72, 3.22)	2.57 (1.24, 5.33)	0.005
Model 2	1	1.24 (0.52, 2.92)	1.60 (0.71, 3.61)	2.50 (1.13, 5.53)	0.017
Model 3	1	1.23 (0.51, 2.95)	1.62 (0.71, 3.71)	2.38 (1.06, 5.37)	0.028
Model 4	1	1.34 (0.54, 3.31)	1.46 (0.62, 3.44)	2.20 (0.94, 5.15)	0.076
Model 5	1	1.23 (0.48, 3.13)	1.32 (0.55, 3.14)	1.86 (0.78, 4.43)	0.165

AST, aspartate transaminase; ALT, alanine transaminase; SBP, systolic blood pressure; hsCRP, high-sensitivity C-reactive protein; HDL, high-density lipoprotein; LDL, low-density lipoprotein. Model 1: Adjusting for age and sex. Model 2: age, sex, AST, ALT and total bilirubin. Model 3: age, sex, AST, ALT, total bilirubin, SBP, glucose and hsCRP. Model 4: age, sex, AST, ALT, total bilirubin, SBP, glucose, hsCRP, triglyceride, HDL and LDL cholesterol. Model 5: age, sex, AST, ALT, total bilirubin, SBP, glucose, hsCRP, triglyceride, HDL, LDL cholesterol and waist circumference. *P*-values represent the results of multiple logistic regression analysis.

## Discussion

The present study shows that circulating LECT2, which has been recently redefined as a hepatokine, is significantly higher in subjects with NAFLD and/or metabolic syndrome compared to those without NAFLD and metabolic syndrome. Furthermore, plasma LECT2 levels had significant positive correlations with various metabolic risk factors including abdominal obesity, adverse lipid profiles, and low-grade systemic inflammatory status.

Inflamed visceral adipose tissue is the main source of an elevated flux of free fatty acids into the portal vein for direct transport to the liver and subsequent hepatic fat accumulation [16]. This means that NAFLD is a sensitive marker for pathological accumulation of visceral fat. Meanwhile, hepatic steatosis itself leads to endoplasmic reticulum (ER) stress, leading to activation JNK and nuclear factor κB (NFκB), two major regulators of inflammatory pathways that exacerbate insulin resistance both locally in the liver and systemically [2]. Therefore, the liver can function as an inducer of systemic inflammation as well as a target of various inflammatory reactions that occur with dysfunctional adipose tissue. Hepatokines, which are exclusively secreted by the liver, directly affect energy metabolism by modulating insulin signaling and inflammatory cascades [17]. In a recent clinical trial, treatment with an analog of fibroblast growth factor 21 (FGF21), a representative hepatokine, produced significant improvements in dyslipidemia in humans with obesity and type 2 diabetes [18]. Previously, we showed a significant decrease in circulating fetuin-A level, which was the first hepatokine discovered, after 12 weeks of caloric restriction. This decrease in fetuin-A was accompanied by improvements in visceral fat area, blood pressure, lipid profiles, and glucose levels [19]. These results suggest that the changes in circulating hepatokines might be associated with the beneficial effects of caloric restriction. Therefore, discovery of novel hepatokines may provide profound insight and promising therapeutic targets for NAFLD-related metabolic disturbances including type 2 diabetes and CVD.

LECT2 is a recently discovered hepatokine that contributes to the development of skeletal muscle insulin resistance in obesity [10]. In a study by Lan *et al*., a treadmill running challenge revealed that muscle endurance was significantly higher in LECT2^-/-^ mice [10]. In addition, C2C12 myocytes transfected with the LECT2 vector showed a significant decrease in insulin-stimulated Akt phosphorylation and an increase in basal JNK phosphorylation [10]. Furthermore, a double knockdown of JNK1 and JNK2 rescued the cells from the inhibitory effects of LECT2 on insulin signaling [10]. Likewise, we previously showed that the level of phosphorylated JNK was significantly increased by LECT2 treatment in HUVEC and THP-1 cells [12]. Considering that the JNK signaling pathway plays a crucial role in diverse metabolic responses to inflammation [20], LECT2, which activates the JNK pathway, might be involved in the various metabolic disturbances in humans. However, very few studies have explored the clinical relevance of circulating LECT2 levels in humans.

Lan *et al*. [10] found a significant positive correlation between plasma LECT2 levels and BMI, waist circumference, and homeostasis model assessment of insulin resistance (HOMA-IR). Also, Okumura *et al*. [21] used receiver operating characteristics (ROC) curve analysis and showed that plasma LECT2 levels efficiently discriminated between subjects with and without fatty livers. However, the relationship between LECT2 and NAFLD that they reported did not adjust for potential confounding factors. In the present study, subjects with NAFLD had significantly higher circulating LECT2 levels when compared to subjects without NAFLD. Furthermore, the OR for NAFLD was 2.38 (95% CI, 1.06–5.37) in the highest quartile of serum LECT2 levels when compared to that of the lowest quartile even after adjusting for other covariates including age, sex, AST, ALT, total bilirubin, systolic blood pressure, serum glucose and hsCRP levels. However, the statistical significance was attenuated after adjusting for serum triglyceride, HDL-C, LDL-C levels and waist circumference, suggesting that LECT2 might be associated with the development of NAFLD through mediation of dyslipidemia and abdominal obesity. Indeed, the present study revealed that LECT2 levels are significantly higher in patients with metabolic syndrome compared to those without metabolic syndrome.

Previously, we demonstrated that LECT2 increased mammalian target of rapamycin (mTOR) phosphorylation, sterol regulatory element-binding protein (SREBP)-1 cleavage, lipid accumulation, and insulin resistance in HepG2 cells [11]. Furthermore, in our previous research, LECT2 treatment efficiently increased the expression of adhesion molecules and pro-inflammatory cytokines in HUVECs and THP-1 cells [12], suggesting the possibility that LECT2 might directly mediate atherosclerotic inflammatory reactions in human endothelial cells. In fact, monocyte chemoattractant protein-1 (MCP-1), a C-C chemokine, and its receptor, C-C chemokine receptor-2 (CCR2), are known to play an important role in atherogenesis [22,23]. Furthermore, accumulated evidence has demonstrated that NAFLD is an independent risk factor for CVD [24]. In a study of 15,913 Korean adults, greater severity of NAFLD showed a higher correlation with estimated 10-year CVD risk [25]. However, the present clinical study showed no significant association between circulating LECT2 levels and subclinical atherosclerosis measured by CIMT and baPWV values. The exact reason for this discrepancy between animal and human studies is not clear, and further study is needed to explore the relationship between LECT2 and atherosclerosis. Instead, we found that plasma LECT2 levels had a significant positive correlation with circulating hsCRP levels, a representative systemic inflammatory marker in subjects with NAFLD, but not in those without NAFLD. Many previous studies have shown that increased hsCRP levels are associated with an increased risk of CV events even after adjustment for traditional risk factors [26]. Similarly, there were significant correlations between circulating LECT2 levels and various metabolic risk factors in subjects with NAFLD, but not in subjects without NAFLD. Lan *et al*. [10] showed that LECT2 plays a major role in the regulation of insulin sensitivity under excess caloric conditions, but not under restricted caloric conditions. Therefore, LECT2 may be associated with metabolic disturbances and unfavorable clinical consequences in individuals with obesity and NAFLD.

The present study has several limitations. First, due to the inherent limitations of a cross-sectional study design, no definite conclusions about causality between circulating LECT2 levels and the development of NAFLD or metabolic syndrome can be drawn. Secondly, only relatively healthy Asian men and women were enrolled in this study, so the results of the current study should be further evaluated in other ethnic populations. Thirdly, we diagnosed NAFLD using ultrasonography. Although liver biopsy is a gold standard for the diagnosis of NAFLD, it is not feasible in epidemiologic studies including large numbers of participants due to its invasiveness. Lastly, this study could not clarify the functional mechanisms of LECT2 on the inflammatory process.

In conclusion, circulating LECT2 levels were significantly associated with NAFLD and metabolic syndrome mainly by mediating dyslipidemia and abdominal obesity. Further large–scaled prospective studies and the experimental studies to explore the mechanistic pathways and functional roles of LECT2 as a novel hepatokine are warranted.

## Supporting information

S1 Table Raw data set of this study. (XLS)

## Abbreviations

ALT: alanine aminotransferase
AMPK: AMP-activated protein kinase
AST: aspartate aminotransferase
baPWV: brachial ankle pulse wave velocity
BMI: body mass index
CCR2: C-C chemokine receptor-2
CIMT: carotid intima-media thickness
CVD: cardiovascular disease
ER: endoplasmic reticulum
FGF21: fibroblast growth factor 21
HCC: hepatocellular carcinoma
HOMA-IR: homeostasis model assessment of insulin resistance
hsCRP: high sensitivity C-reactive protein
HUVECs: human umbilical vein endothelial cells
JNK: Jun NH2-terminal kinase
LECT2: leukocyte cell-derived chemotaxin 2
MCP-1: monocyte chemoattractant protein-1
MetS: metabolic syndrome
mTOR: mammalian target of rapamycin
NAFLD: non-alcoholic fatty liver disease
NFκB: nuclear factor κB
PAI-: plasminogen activator inhibitor-1
ROC: receiver operating characteristics
SREBP: sterol regulatory element-binding protein
WBC: white blood cell
